# Accuracy assessment of a potential clinical use of navigation-guided intra-operative liver metastasis brachytherapy—a planning study

**DOI:** 10.1007/s00066-018-1334-y

**Published:** 2018-07-18

**Authors:** E. Herrmann, D. Terribilini, P. Manser, M. K. Fix, G. Toporek, D. Candinas, S. Weber, D. M. Aebersold, K. Loessl

**Affiliations:** 1 Department of Radiation Oncology, Inselspital, Bern University Hospital, University of Bern, Freiburgstr., 3010 Bern, Switzerland; 2 Division of Medical Radiation Physics and Department of Radiation Oncology, Inselspital, Bern University Hospital, University of Bern, Bern, Switzerland; 3 ARTORG Center for Biomedical Engineering Research, University of Bern, Bern, Switzerland; 4 Department of Visceral Surgery and Medicine, Inselspital, Berne University Hospital, University of Berne, Bern, Switzerland

**Keywords:** Liver, Brachytherapy, Image guidance, Radiotherapy, Liver metastases, Leber, Brachytherapie, Bildführung, Radiotherapie, Lebermetastasen

## Abstract

For patients with inoperable liver metastases, intra-operative liver high dose-rate brachytherapy (HDR-BT) is a promising technology enabling delivery of a high radiation dose to the tumor, while sparing healthy tissue. Liver brachytherapy has been described in the literature as safe and effective for the treatment of primary or secondary hepatic malignancies. It is preferred over other ablative techniques for lesions that are either larger than 4 cm or located in close proximity to large vessels or the common bile duct. In contrast to external beam radiation techniques, organ movements do not affect the size of the irradiated volume in intra-operative HDR-BT and new technical solutions exist to support image guidance for intra-operative HDR-BT. We have retrospectively analyzed anonymized CT datasets of 5 patients who underwent open liver surgery (resection and/or ablation) in order to test whether the accuracy of a new image-guidance method specifically adapted for intra-operative HDR-BT is high enough to use it in similar situations and whether patients could potentially benefit from navigation-guided intra-operative needle placement for liver HDR-BT.

## Zusammenfassung

Für Patienten mit inoperablen Lebermetastasen ist die intraoperative Hochdosis-Brachytherapie (HDR-BT) eine vielversprechende Technologie, die es ermöglicht, eine hohe Strahlendosis auf den Tumor zu liefern, während das umliegende gesunde Gewebe geschont wird. Die Leber-BT wurde in der Literatur als sicher und wirksam für die Behandlung von primären oder sekundären Tumoren in der Leber beschrieben. Sie ist für Läsionen mit einer  Größe > 4 cm oder in unmittelbarer Nähe zu großen Gefäßen sowie in engem Kontakt mit dem zentralen Gallengang gegenüber anderen ablativen Verfahren zu bevorzugen. Im Gegensatz zu den externen Strahlentherapietechniken wirken sich bei der intraoperativen HDR-BT Organbewegungen nicht auf die Größe des bestrahlten Volumens aus. Neue bildgestützte technische Lösungen existieren, um die intraoperative Applikatoreinlage für die HDR-BT zu unterstützen. Wir haben retrospektiv anonymisierte Computertomographiedatensätze von 5 Patienten analysiert, welche eine offene Leberoperation erhalten haben (Resektion und/oder Ablation). Hierbei testeten wir, ob die Genauigkeit der speziell für die intraoperative HDR-BT angepasste Navigation ausreicht, um sie in vergleichbaren Situationen anzuwenden, und ob Patienten möglicherweise von der neuen Bildführungsmethode zur intraoperativen Nadelplatzierung bei der Leber-HDR-BT profitieren könnten.

## Introduction

Colorectal cancer (CRC) is the most frequent primary site to give rise to metastatic liver disease. The role of local therapy has been most thoroughly evaluated in CRC hepatic metastases [1]. Surgical resection has become the preferred treatment for these patients, since it can provide cure of disease and is associated with 5‑year survival rates in the range of 25–47% [2–5]. However, due to associated co-morbidities, non-resectable extrahepatic disease, or multiple liver lesions with inadequate residual functioning liver parenchyma after surgery, 70–80% of patients with colorectal liver metastases are not candidates for resection [6]. This has encouraged the use of alternative surgical approaches, such as radiofrequency and microwave tumor ablation, as well as cryotherapy and other ablative techniques [7]. For small tumors, radiofrequency ablation (RF) or microwave ablation (MWA) achieve excellent local control [8]. However, high local recurrence rates of up to 39% [9], particularly in tumors > 3 cm and tumors adjacent to large vessels [8, 10], remain a challenge. There is growing interest in applying radiotherapy to hepatic malignancies. A decade ago, image-guided high dose-rate brachytherapy (HDR-BT) of liver tumors was introduced [11] as a local radio-ablative technique, in which an iridium-192 source is temporarily inserted into the tumor through catheters placed under image guidance [12]. HDR-BT is preferred over other thermal ablative techniques for lesions > 4 cm in size or located near large vessels as well as those in close contact with the central bile duct [13, 14, 33, 34]. Any HDR-BT intervention within the liver requires an in-depth three-dimensional (3D) understanding of the organ’s internal vascular and biliary anatomy. To optimize the treatment outcomes, it is important that these procedures result in accurate positioning of the needle to achieve complete tumor dose coverage and minimize the probability of damage to surrounding normal tissue. Moreover, there might be organs at risk (OAR)—such as the hepatic arteries, portal veins, and bile ducts—in the proximity of the needle path that imperatively should not be touched. For surgical interventions, the introduction of pre-operative 3D imaging data from computer tomography (CT) or magnetic resonance imaging (MRI) in the early 1990s enabled surgeons to virtually plan and evaluate surgical strategies prior to the actual surgery using the available imaging data [36]. Clinically applicable surgical planning solutions are nowadays available [37]. Computer-assisted open liver surgery has now been available for 10 years, but only recently were image guidance systems specifically optimized for hepatopancreaticobiliary (HPB) surgery [15, 38]. The missing link between virtual surgery planning and pre-operative evaluation is intra-operative navigation support for the real-time reproduction of the virtually planned surgical intervention, with the goal of increased precision of the planned intervention. Such a navigation support consisting of an optically based instrument guidance system is already in use in our hospital for open liver surgery [15] and MWA [16], and may also facilitate the intra-operative and CT-guided application of HDR-BT.

The aim of the current study is twofold: to translate the knowledge from virtual planning and evaluation of liver surgeries from pre-operative 3D imaging and intra-operative navigation support into the world of HDR-BT. The goal is to investigate the feasibility and accuracy of a navigation support for the placement of brachytherapy needles for liver HDR-BT in a phantom, simulating liver metastases with copper wires. In a second step, we evaluate how to translate the yielded accuracy results from the phantom study into a clinical setting, along with the clinical exploration of such an approach based on HDR-BT plans.

## Materials and methods

Experiments carried out in this work are twofold and consist of the investigation (1) of the feasibility of using a previously available stereotactic image-guidance system for CT-based interventions for the placement of HDR-BT applicators in a realistic phantom in which liver metastases were mimicked using copper wires. We have measured the accuracy of navigated needle placement and compared it with the accuracy of historical experience of free-hand needle placement in a phantom and translated these results into a clinical setting. Subsequently, (2) we have retrospectively created HDR-BT plans of patients with colorectal liver metastasis by taking the measurement results from the phantom experiment into account to explore what impact a potential inaccuracy of the needle placement with a navigation system would have on HDR-BT dosimetry.

### Feasibility of image-guided HDR-BT placement

#### System overview

**Fig. 1:**
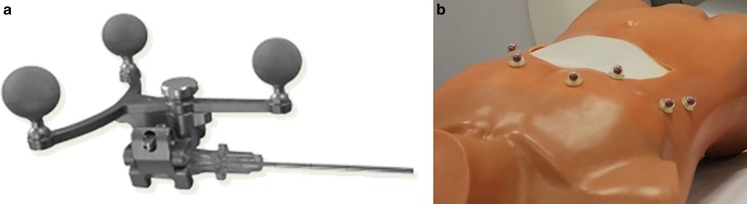
Markers for needle (**a**) and patient (**b**) tracking

A previously available navigation system for percutaneous interventional procedures (CAS-One IR, CAScination AG, Bern, Switzerland; [17]) was adapted to investigate the experimental feasibility of brachytherapy needle placement during intra-operative HDR-BT. This system combines planning of the needle trajectories in CT data and the subsequent placement of these needles (i.e., ablation applicators) under real-time guidance. It tracks both the patient and needle placement process through an optical position measurement system (NDI Vicra, Northern Digital, Canada). Marker shields with retro-reflective passive markers can be adapted and attached to a variety of interventional tools (Fig. 1a). The system provides tracking of the patient’s respiratory motion through retro-reflective markers, which are attached to the patient’s skin prior to the acquisition of a planning CT (Fig. 1b). Their positions are then automatically detected in the CT slices [18]. Per tracking cycle (20 Hz), the optically detected marker positions are co-registered to the image data using a rigid point-based registration algorithm. The resulting fiducial registration error (FRE) is computed and serves as a correlate of the available deformation of the patient’s abdomen due to respiratory motion.

**Fig. 2:**
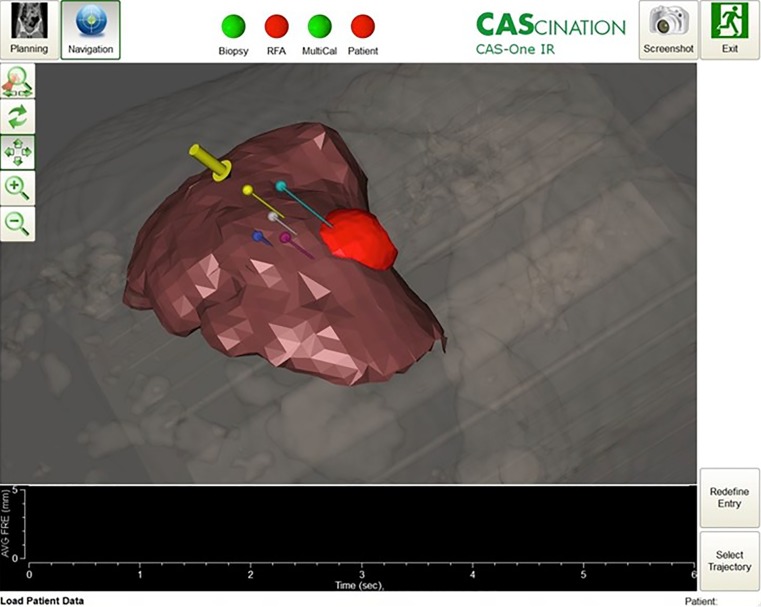
When the needle is far away from the planned entry, the system displays a reconstruction of the treatment plan with the whole organ, an isodose volume (in *red*), and the planned needle trajectories (in *dark blue, purple, grey, yellow* and *light blue*). The *yellow* trajectory is currently activated

**Fig. 3:**
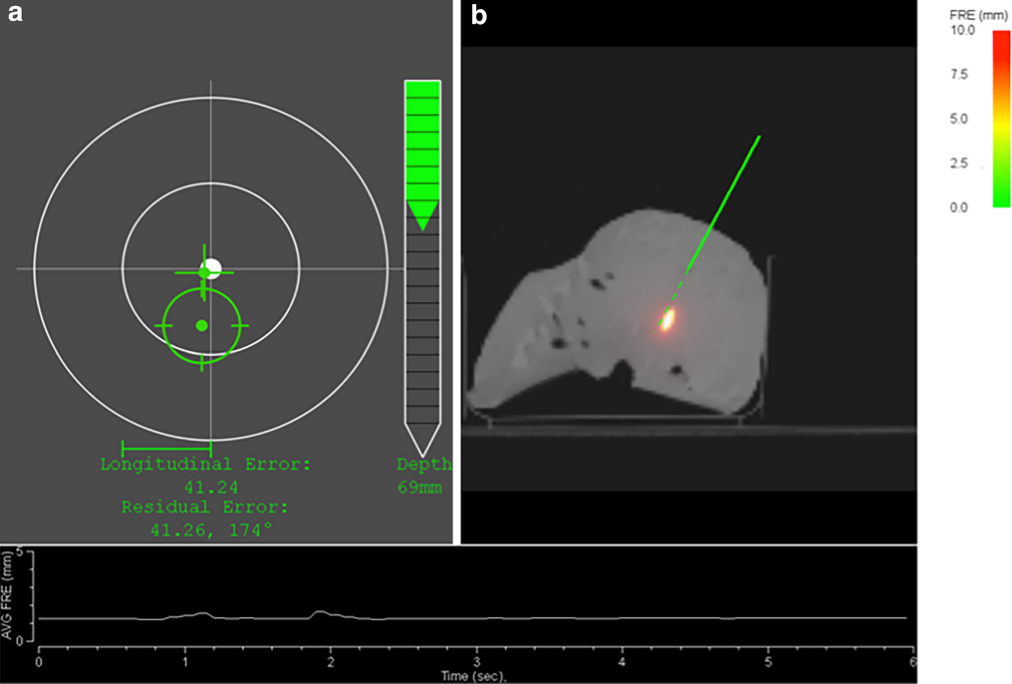
When the needle is close to the planned entry, the system displays a cross-hair viewer (**a**), where the needle tip is represented by a *cross* and the needle shaft by a *circle*. A depth bar guides the user towards the target. Displayed in **b** is a resliced CT, showing the current path of the needle. The curve at the bottom of the screen displays the amplitude of patient deformation

The system allows for image-based identification of the target and organs at risk as well as the planning of the planned needle trajectories (Fig. 2). Subsequently, the needle placement is carried out using the system’s integrated guiding functionality consisting of an abstract cross-hair view as well as an image-based guiding viewer (Fig. 3). The user initially aligns the needle tip relative the prospective entry, and adjusts the needle’s correct longitudinal alignment. Once alignment and orientation are according to the plan, the needle can be advanced towards the target. The system was adapted to enable the experiments described in this work.

#### Image-guided placement of HDR-BT applicators

**Fig. 4:**
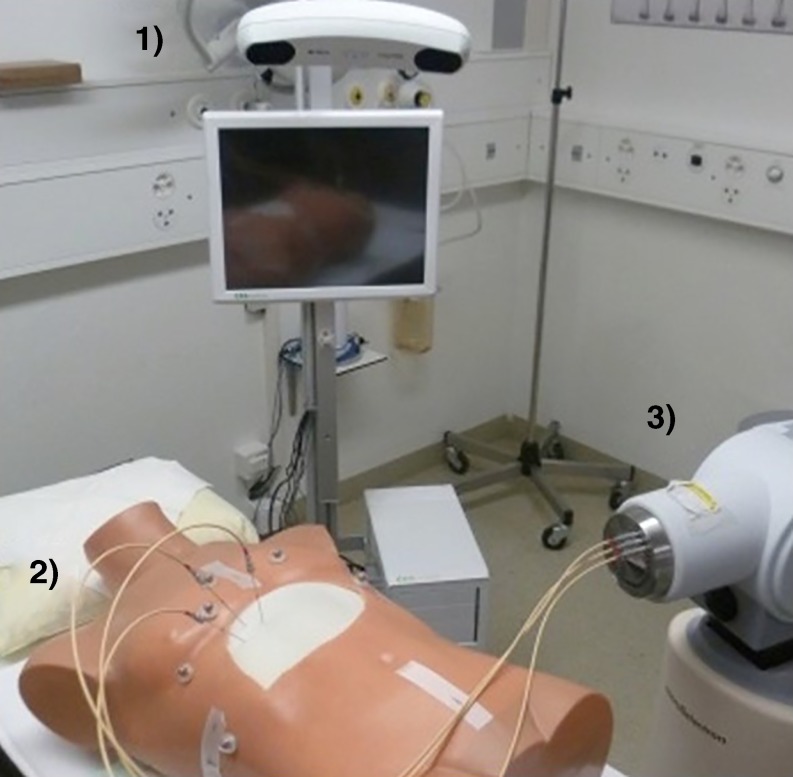
Experimental setup with the navigation system (*1*), the phantom (*2*), and the remote afterloader (*3*), *HDR* high-dose rate

The feasibility of the workflow and the accuracy of the system were evaluated by performing three punctures on a realistic phantom. This phantom was made of a gelatin-filled plastic bag placed inside a 1:1 scale plastic torso (Fig. 1b). Liver metastases within the phantom were mimicked using copper wires. Five copper wires of a diameter of 1.6 mm were inserted inside the gelatin-filled plastic bag and served as targets. Six retro-reflective markers were glued to the phantom surface for deformation tracking and a pre-interventional CT scan of the phantom was acquired. The placement experiment was carried out as follows:

1. In the first step of the evaluation, a planning target volume (PTV) containing one of the copper wires was manually delineated in the CT data.
2. A treatment plan ensuring a PTV coverage of 97% with three needles was designed.
3. Up to three punctures were performed along the planned trajectories (Fig. 4).
4. A post-interventional control CT was acquired after needle insertion in which the position of the inserted needles was reconstructed and the actual dose to the PTV re-calculated.

#### Evaluation of the image guidance system

In order to evaluate the feasibility of the guidance approach, the accuracy of the system, defined as target positioning error (TPE), was assessed. It was computed for each needle as the distance between the planned and the achieved needle tip position. TPE was separated into a lateral component (along the needle trajectory) and the angular component. The overall accuracy of the approach was also investigated by computing the dose delivered to the PTV and comparing it to the intended dose.

### Retrospective suitability assessment for HDR-BT

**Fig. 5:**
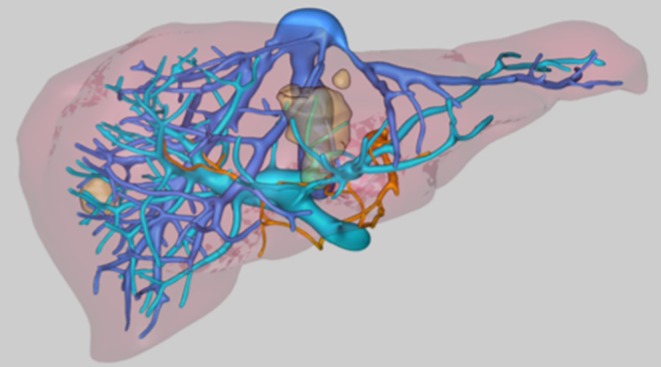
Example of the three-dimensional workup of an individual dataset. The image shows the hepatic veins (*blue*), the portal vein (*blue-green*) and the vessel of interest (*orange*). The metastases are seen a brown masses, distributed in both liver lobes.

Retrospectively, available anonymized multiphasic, contrast-enhanced CT scans of the liver image data and corresponding 3D workup (MeVis Medical Solutions AG, Germany; Fig. 5) of 5 patients with colorectal liver metastases previously treated with a combination of surgical liver resection and/or ablation (treated in our hospital between 06/2011 and 06/2012) were selected for use in this analysis (see Table 1 for a description of the selected datasets). More specifically, anonymized image data of those patients who met the following criteria were identified: eligibility for surgery, hepatic tumor load < 40% of total liver volume or less than 6 liver metastases, ≥1 lesion located next to a critical structure. Exclusion criteria for this study were >6 metastases and presence of primary liver tumors.

**Table 1:** Description of the selected study group

ID	No. of lesions	Volume (ml)	Location of Lesions
Patient 1	4	17	Segments IV, V
Patient 2	5	52	Segments III, IV, VII, VIII
Patient 3	1	24	Segment VIII
Patient 4	4	3	Segments V, VI, VII, VIII
Patient 5	3	12	Segments III, IV, VII

#### HDR-BT treatment planning

For the selected patients, HDR-BT plans were generated within the treatment planning system Masterplan v4.1 (Nucletron, Elekta AB, Stockholm, Sweden). Contouring and 3D dose optimization were performed on patients’ pre-interventional CT datasets with slice thicknesses of 1 mm. For each patient, gross target volumes (GTV) were defined and manually contoured. From each GTV, a clinical target volume (CTV) was created by adding a 0.5 mm margin. Since the setup errors in brachytherapy can be assumed to be negligible, CTV corresponds to PTV. In general, for PTVs up to a diameter of 2 cm, one applicator was used, while for PTVs larger than 2 cm, two to three applicators were employed to improve conformity of the dose distribution. Dwell positions and weights of each applicator were modified until dose–volume constraints were optimally achieved. Prescription dose (PD) of the CT plans was 20 Gy, with the dosimetric goal to cover at least 90% of the PTV with the PD (V_100%_ PTV ≥ 90%). For the resulting treatment plans, dose–volume histogram (DVH) parameters for PTV and OARs were evaluated.

## Results

### Feasibility of image guidance

**Fig. 6:**
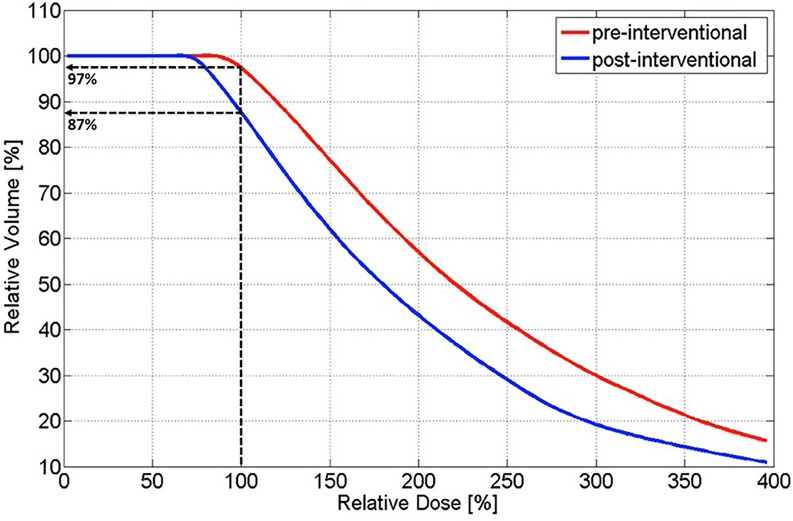
Dose–volume histograms of planned and achieved dose distributions

The achieved TPE with the navigation system was 4.2 mm ± 1.2 mm, with an average lateral error of 0.5 mm ± 0.1 mm and an average angular error of 2.6° ± 0.4°. In the pre-interventional planning, 97% of the PTV received 100% of the dose (V_100%_). After the insertion of three brachytherapy needles with the navigation system, the post-interventional plan shows that V_100%_ decreased to 87% (Fig. 6).

### Suitability of treatment planning

Over the five selected patient plans, 13 PTVs ranging between 2.2 cm^3^ and 65.3 cm^3^ were contoured. The average coverage (V_100%_) of the 13 PTVs was 94.2 ± 2.9%. For a PD of 20 Gy per fraction, an average median dose of 2.8 ± 1.1 Gy to the normal liver (Liver-PTV) was calculated. For the same PD and a typical source strength of 4 x 10^4^ cGy x cm^2^/h, the total treatment times last between 8 and 32 min.

Over the five patient plans, the conformal index (COIN) according to [19] was 0.55 ± 0.03.

## Discussion and conclusion

General surgical stereotactic systems have been known since the early 1990s and enable surgeons to virtually plan and evaluate surgical strategies prior to the actual surgery using the available imaging data from CT or MR imaging. Immobile skeletal structures such as the rigid neurocranium provided the optimal setting for precise image guidance. Therefore, initial experiences with computer-assisted and image-guided surgery were mainly gained from rigid structures and applied in neurosurgery, complex craniofacial surgery, and orthopedic surgery [36]. In contrast, the liver parenchyma is exposed to constant breathing motion and organ deformation. Only more recently for HPB surgery have first image guidance systems taken the complexity of the liver itself into account to a sufficient level to permit their application in daily clinical practice [38, 39]. Nevertheless, bridging the gap between virtual pre-operative intervention planning and intra-operative navigation support for the real-time reproduction of the virtually planned intervention remains a challenge. Deformations of the liver of up to 20 mm have been detected when compared to a pre-operatively acquired 3D tomographic image [40]. Thus, the fundamental challenge in image guidance in liver interventions is a correct and real-time referencing of the pre-operatively acquired image data (3D- and 4D-/contrast-enhanced/CT or MRI) with the actual intra-operative situation. Such a navigational support of an optically based instrument guidance system is already in use in our hospital for open liver surgery [15] and MWA [16]. Currently, such image-guided navigation systems are not used for the placement of applicators for liver HDR-BT. In the present study, we demonstrate the potential use of this available image-guidance technology for interventional procedures with specific adaptions to accommodate the placement of HDR-BT applicators.

**Fig. 7:**
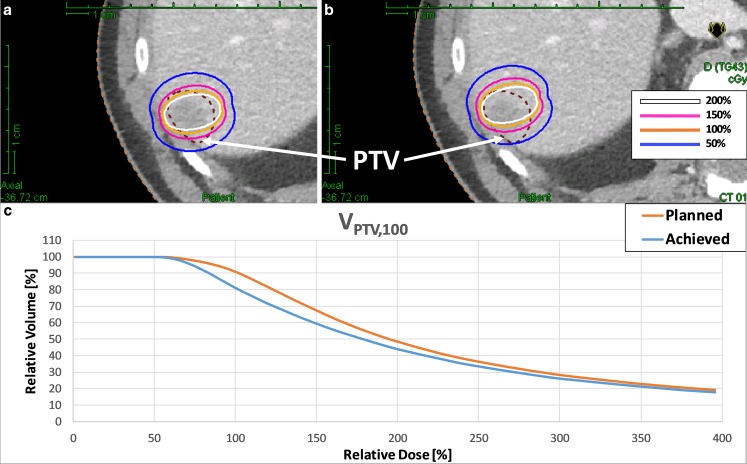
Example of planned (**a**) and achieved (**b**) dose distributions in a patient with a PTV volume of 6.6 ml (median volume over the analyzed patient collective). The achieved dose distribution was simulated applying a TPE of 4.2 mm ± 1.2 mm. **c** Dose–volume histograms of planned and achieved dose distributions

Initial studies of intra-operative liver brachytherapy first described the technique in the 1980s [20–22]. At that time, applicator placement was guided either by manual palpation or sonographic monitoring [22]. In such a situation, neither adequate coverage of the target volume nor exact sparing of adjacent critical structures could be guaranteed. A decade ago, image-guided brachytherapy of liver tumors was introduced [11], in which an iridium-192 source is temporarily inserted into the tumor through catheters placed under CT image guidance [11, 23–25]. In the present study, we demonstrate an effective geometric accuracy of 4.2 mm ± 1.2 mm for the placement of the HDR-BT applicators using such an available image-guidance technology for interventional procedures, with a potential degrading of V_100%_ in the PTV from 97 to 87% (Fig. 7). In a subsequent study at our department [26], we analyzed the dosimetric impact of geometric uncertainties (i.e., tumor size, shape, number of lesions, needle length and position, number of needles as well as OAR and target volume coverage) in navigated HDR-BT for liver tumors by applying different shifts (≤5 mm and ≤1 cm) to a known dose distribution. It was found that for small PTVs below 2 cm, the deviations for dose coverage and dose conformity can be up to 50%. The effect depends on the number as well as on the placement and arrangement of needles and decreases with increasing tumor size. For larger PTVs, the reduction in dose coverage for a ≤5 mm shift is between 5 and 10%. It was further concluded that where no other treatment modality is available, such a navigation-guidance method for intra-operative HDR-BT seems to be promising. However, specific inclusion/exclusion criteria for patient selection for navigated intra-operative HDR-BT remain unclear at this stage.

In clinical practice, several possibilities exist to overcome these uncertainties. Using more than one applicator for a liver brachytherapy plan enables better coverage of complex-shaped tumor volumes, as well as better protection of the healthy surrounding tissue with its rapid dose fall-off outside the target lesion. It also increases the robustness of the plan. Additionally, the non-optimal placement of the brachytherapy needle can be compensated by a corresponding adjustment of the dwell time of the source within the target volume. With these various adjustable elements for planning, the minimum target dose at the tumor border can be achieved with no compromise in target coverage. Since the goal in liver brachytherapy is to cause necrosis in the tumor, dose heterogeneity within the target volume is of no concern and no maximum dose limit exists [27, 32].

The assessment of the present datasets indicates the feasibility of navigated intra-operative HDR-BT. The method is potentially feasible for all these patients with either a hepatic tumor load below 40% of the total liver volume or less than six liver metastases larger than 4 cm. For the selected datasets, acceptable doses to the normal liver as well as acceptable treatment times have been observed. The risk of harming blood vessels by placing applicators in liver brachytherapy is potentially mitigated through the use of the described guidance method by reducing the need for re-placement due to inaccuracy.

In clinical practice we often face complex settings in patients with CRC hepatic metastases, such as multiple, synchronous, bi-lobar, and large hepatic lesions that are located near critical structures or blood vessels. Combining this navigation-guided intraoperative HDR-BT with surgery and MWA also opens new opportunities for a combined treatment approach at the same time. Such a computer-navigated system may facilitate the application of HDR-BT for the placement of BT applicators and planning of liver HDR-BT also in situations where HDR-BT was not initially planned to be part of the intervention.

With regards to the dose prescription, in the present study we prescribed 20 Gy to at least 90% of the PTV. Previous studies of intraoperative brachytherapy for liver cancers proposed that tumor-enclosing isodoses of 15 to 30 Gy should be used [20–22, 28]. In a prospective randomized trial of 73 patients with 199 colorectal liver metastases, Ricke et al. [29] evaluated the minimal dose levels enclosing the CTV of 15, 20, and 25 Gy. They found a dose dependency for local control if the minimum dose in the target exceeded 20.4 Gy. In a multivariate analysis, lesion size (range of tumors 1–13.5 cm) had no impact on local control. However, in tumors larger than 8 cm, excessive irradiation time and unpredictable risks aggravated the delivery of >20 Gy in a single fraction. Nevertheless, these data show that lesions up to a diameter of 8 cm can be safely treated with a single fraction of 20 Gy applied by HDR-BT.

There are several limitations to this feasibility study. First, any conclusions revealed here are hypothesis-generating and, as such, will need to be validated within a prospective study. Several open questions for intraoperative navigation-guided HDR-BT remain: it is unclear how many applicators need to be placed for lesions > 8 cm and what conformity should be achieved, especially in bigger lesions. With only one applicator, dose conformity might be too low. More applicators might provide a better conformity of dose distribution. We suggest the number of applicators should depend on the size of the lesion. Also, dose constraints for liver HDR-BT are unclear. In their studies, Ricke et al. [28] used a maximum dose exposure of 5 Gy to maximally two thirds of the liver volume as a liver constraint. In their 2005 published study [25] on dose–response relationship for small volumes of liver parenchyma after single-fraction HDR irradiation, they used, after brachytherapy, a hepatocyte-specific contrast agent media uptake (Gd-BOPTA) for liver MRI as a surrogate for liver function. They found that the lowest threshold dose to impair hepatocyte function was 9.9 Gy (standard deviation 2.3 Gy) at 6 weeks after irradiation with repair ongoing until 3 months. After single-fraction stereotactic body radiotherapy (SBRT) to liver malignancies, Herfarth et al. [30] observed a focal reaction after contrast CT at a dose minimum of 13.7 Gy (range 8.9–19.2), which is consistent with the experience in brachytherapy. In the current study, we have used the dose constraints for stereotactic body radiation therapy (SBRT) according to Grimm et al. [31], with which technique also high doses of single fractions can be applied [35].

Since the technology of this system was initially developed for intra-operative support of liver resection and MWA, we have used this clinical setting to evaluate the potential benefit of the use of such a navigation system for liver BT. However, the use of this stereotactic navigation system opens also new opportunities for the placement of HDR-BT applicators within the liver by percutaneous CT guidance in patients with large, centrally localized liver metastases where no resection or MWA intervention is indicated. It may provide support in pre-interventional treatment planning (i.e., needle path, multi-applicator configuration) and applicator placement (i.e., angle and depth guidance).

In conclusion, we provide evidence for the technical feasibility of navigation-guided intra-operative HDR-BT and propose a set of inclusion criteria for clinical testing.
